# Bibliometric analysis of nanotechnology in spinal cord injury: current status and emerging frontiers

**DOI:** 10.3389/fphar.2024.1473599

**Published:** 2024-12-11

**Authors:** XiaoPeng Gu, SongOu Zhang, WeiHu Ma

**Affiliations:** ^1^ Department of Clinical Medicine, Health Science Center, Ningbo University, Ningbo, Zhejiang, China; ^2^ Department of Orthopedics, Ningbo No. 6 Hospital, Ningbo, Zhejiang, China; ^3^ Department of Orthopedics, Zhoushan Guhechuan Hospital, Zhoushan, Zhejiang, China; ^4^ Department of Orthopedics, Zhoushan Institute of Orthopedics and Traumatology, Zhoushan, Zhejiang, China

**Keywords:** spinal cord injury, nanotechnology, inflammation, bibliometric, CiteSpace

## Abstract

**Objective:**

The objective of this study was to analyze the impact of nanotechnology on the treatment and recovery of spinal cord injury (SCI), a condition that has profound global effects on physical and psychological health.

**Methods:**

We utilized the Web of Science Core Collection to obtain bibliometric data. With the tools such as VOSviewer and CiteSpace, we conducted a comprehensive review of 422 relevant publications to identify research trends and influential works in the field of nanotechnology applied to SCI.

**Results:**

The analysis revealed significant contributions from both China, Sweden and the United States, and pinpointed inflammation, apoptosis, and nano-drug delivery as the primary areas of focus in current research, with emerging trends evident in recent literature.

**Conclusion:**

Nanotechnology hold great potential to revolutionize the treatment of SCI through targeted therapeutics and modulation of pathological processes. This study provided valuable insights into the evolving landscape of SCI research, underscoring the importance of continuous innovation and interdisciplinary collaboration.

## 1 Introduction

SCI denotes the structural and functional impairment of the spinal cord resulting from an external force (McDonald and Sadowsky, 2002). The pathophysiological cascade of SCI encompasses primary and secondary injuries (Eli et al., 2021). The primary injury corresponds to the immediate trauma at the moment of impact, which may include compression, laceration, or severance. Subsequently, secondary injury ensues as a consequence of a complex interplay of biochemical, immunological, and cellular reactions following the primary insult (Fan et al., 2018). These reactions involve inflammation, free radical generation, cellular apoptosis, and glial scar formation, which collectively exacerbate the initial damage. Worldwide, the annual incidence of SCI ranges from 40 to 80 cases per million individuals, predominantly affecting young and middle-aged adults, with a higher prevalence in males than in females. The leading causes of SCI include traffic accidents, falls, sports-related injuries, and acts of violence. Incidence rates vary across different regions and are influenced by factors such as the level of economic development, traffic accident frequency, and lifestyle choices (Kumar et al., 2018; Ding et al., 2022; Liu Y. et al., 2023). The current management of SCI is categorized into acute and rehabilitation phases, tailored to the stage of injury and recovery (Karsy and Hawryluk, 2019). Acute management strategies involve pharmacological interventions, like the administration of corticosteroids (e.g., methylprednisolone) to mitigate inflammation and cellular damage, along with surgical interventions and supportive care, including respiratory support, nutritional therapy, and complication prevention. Rehabilitation protocols encompass physical therapy modalities, such as exercise and balance training, as well as functional electrical stimulation to enhance or restore motor capabilities. Occupational therapy is employed to foster maximum independence in activities of daily living, while psychological therapy provides counseling and support to aid patients in adapting to their altered circumstances (Harvey, 2016). Beyond these established clinical treatments, experimental approaches are being investigated in laboratory settings, including stem cell therapies and biomaterial-based interventions. The application of stem cells in the treatment of spinal cord injury is also of great significance (Venkatesh et al., 2019; Katari et al., 2024). Despite progress in SCI understanding and management, the search for innovative treatments is essential. Nanotechnology is a science that focuses on manipulating and engineering matter at the nanoscale. The definition of nanoparticles usually refers to particles within the nanoscale range (typically between 1 and 1,000 nm). However, in actual applications, a wider range of particles is sometimes included, especially when they exhibit nano-effects. Under a broader definition, nanoparticles can cover a range from 1 nm to several hundred nanometers or even thousands of nanometers. Particles within this range are still considered to possess special properties at the nanoscale. Generally, the smaller the size of the nanoparticle, the more pronounced its surface and quantum effects, particularly when the size is below 100 nm, making its properties even more prominent. Its innovative applications in this realm have brought tremendous potential to the fields of medicine and biotechnology. The classification of nanotechnology can vary in many ways. The simplest method is based on the basic properties of nanoparticles, which can be divided into organic and inorganic nanoparticles. Both types of nanoparticles are widely used in the field of spinal cord injury. Each type has its own characteristics; for instance, inorganic nanoparticles, such as cerium oxide nanoparticles, have significant effects in combating oxidative stress (Behroozi et al., 2022). Organic nanoparticles, such as liposomes and polymer nanoparticles, are more commonly used in drug delivery systems, especially in applications related to targeted drug delivery and cellular regeneration (Wang X. et al., 2021). Based on different applications of nanotechnology, it can also be categorized into drug delivery applications, selective targeting, and imaging/diagnostics. Nanotechnology has been used to develop more effective drug delivery systems. For example, nanoparticles can be used to deliver drugs directly to the site of a disease, reducing damage to healthy tissues and improving therapeutic efficacy. Nanotechnology has also enabled selective targeting applications by designing specific types of nanoparticles that can recognize and bind to particular cell receptors or disease markers. This enhances the precision of drug delivery, allowing treatment to focus on diseased cells while minimizing the impact on healthy cells (Sabourian et al., 2023; Khan et al., 2020). Additionally, applications of nanotechnology in biotechnology include the use of nanosensors for early detection and diagnosis of diseases, and the enhancement of biomedical imaging techniques with nanomaterials, allowing doctors to observe and understand the inside of the human body more accurately. These applications not only improve the precision and efficiency of treatments but also open up new possibilities for personalized medicine and precision therapy, with the potential to greatly improve human healthcare in the future. Nanotechnology offers promising avenues for SCI treatment, highlighted by the field of nanotechnology (Zimmermann et al., 2021; Gao et al., 2021; Bayda et al., 2019; Imran et al., 2023; Alzate-Correa et al., 2022; Santamaría et al., 2023; Saeedi et al., 2019). This discipline explores nanoscale materials, showing significant potential across various sectors, particularly in SCI, where it promises to transform diagnosis, treatment, and rehabilitation strategies.

This bibliometric analysis examines the current trends in nanotechnology for SCI, analyzing research volume, geographic distribution, key researchers, institutions, and citation patterns (Shang et al., 2023). It aims to provide a detailed view of this field’s growth, spotlighting influential studies and emerging directions for future research. This work underscores nanotechnology’s critical role in advancing SCI treatment, and recovery, fostering further innovation and offering hope to those affected by SCI.

## 2 Methods

### 2.1 Data source

In this article, we selected Web of Science Core Collection (WoSCC) as the source of bibliometric data in the field of nanotechnology for SCI research.

### 2.2 Data search strategy

The specific retrieval method has been mentioned in previously published articles (Shang et al., 2023; Zhang et al., 2023; Wang et al., 2024). To put it simply, the data was downloaded and retrieved by two authors on the same day. The search was conducted in the WoSCC database using the following search formula: TS = nano* and [(TS = spinal cord injury) or (TS = spinal cord injuries)]. We only included articles in English and limited the article types to reviews and monographs. A total of 1,367 articles were initially included. Inclusion criteria: Only articles or reviews that focus on nanotechnology in spinal cord injury research will be considered. Papers that only briefly mentioned nanotechnology and spinal cord injury in their abstracts were excluded. Two individuals reviewed the titles, abstracts, and full texts to exclude papers that were not relevant to the topic, resulting in a final inclusion of 422 papers for this study.

### 2.3 Bibliometric analysis

In this article, we utilized five softwares of VOSviewer, CiteSpace, Bibliometrix, SCImago, and GraphPad Prism to conduct bibliometric visual analysis. Specifically, we utilize VOSviewer, CiteSpace, and Bibliometrix to process and analyze the data downloaded from WoSCC. Use VOSviewer to analyze and extract information and build visualization maps of countries, institutions, authors, keywords. Use CiteSpace to construct a keyword busrt visualization graph. Use Bibliometrix analysis to extract information on the number of publications and citations. Use SCImago to build a visualization map of collaborations between countries, institutions, and authors. Use GraphPad Prism software to build visualization graphs of the number of publications and citations. The specific procedures were described in previous articles (Zhang et al., 2023; Wang et al., 2024).

## 3 Results

### 3.1 Publication outputs and trends

**FIGURE 1:**
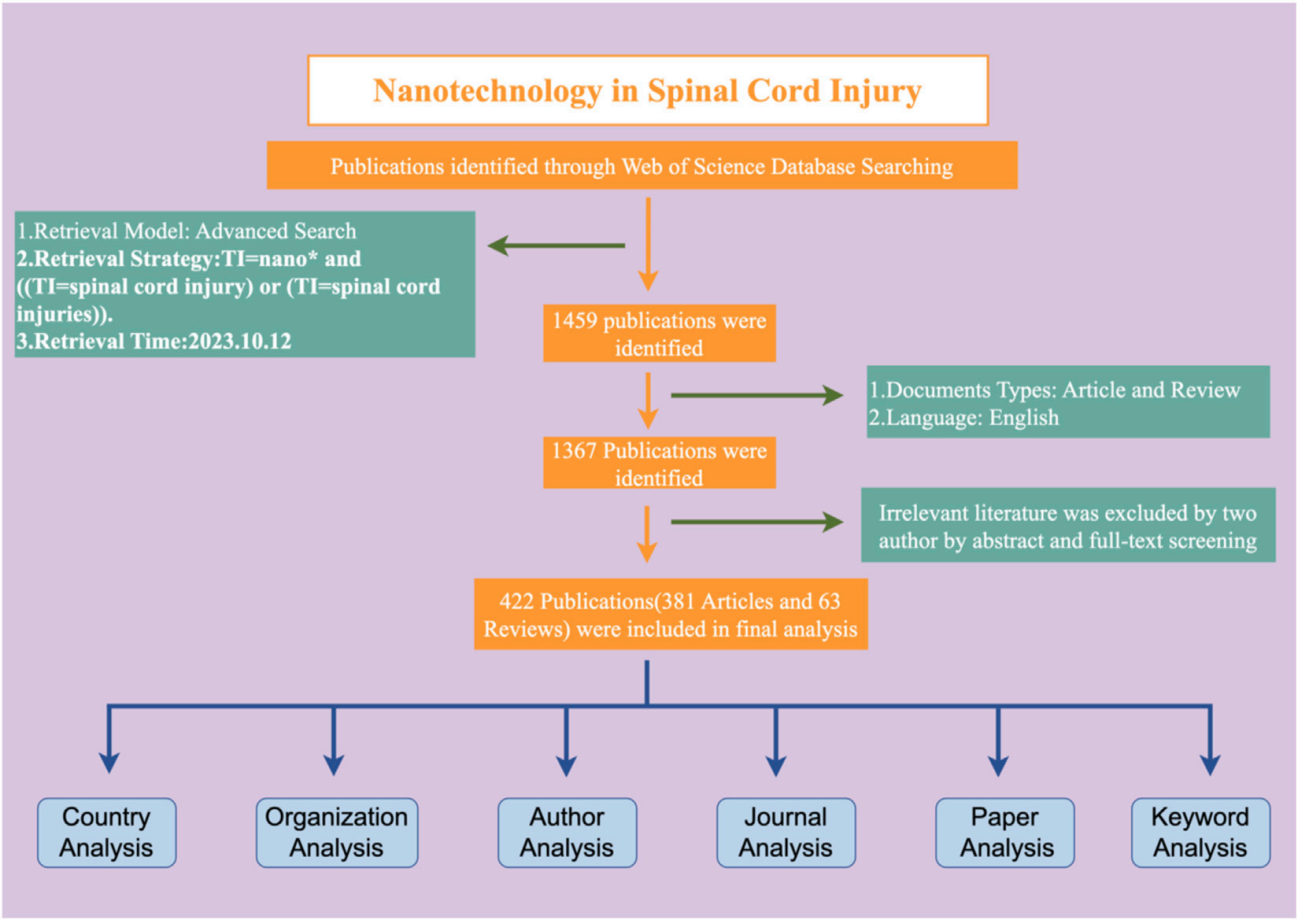
Publication screening flowchart.

**FIGURE 2:**
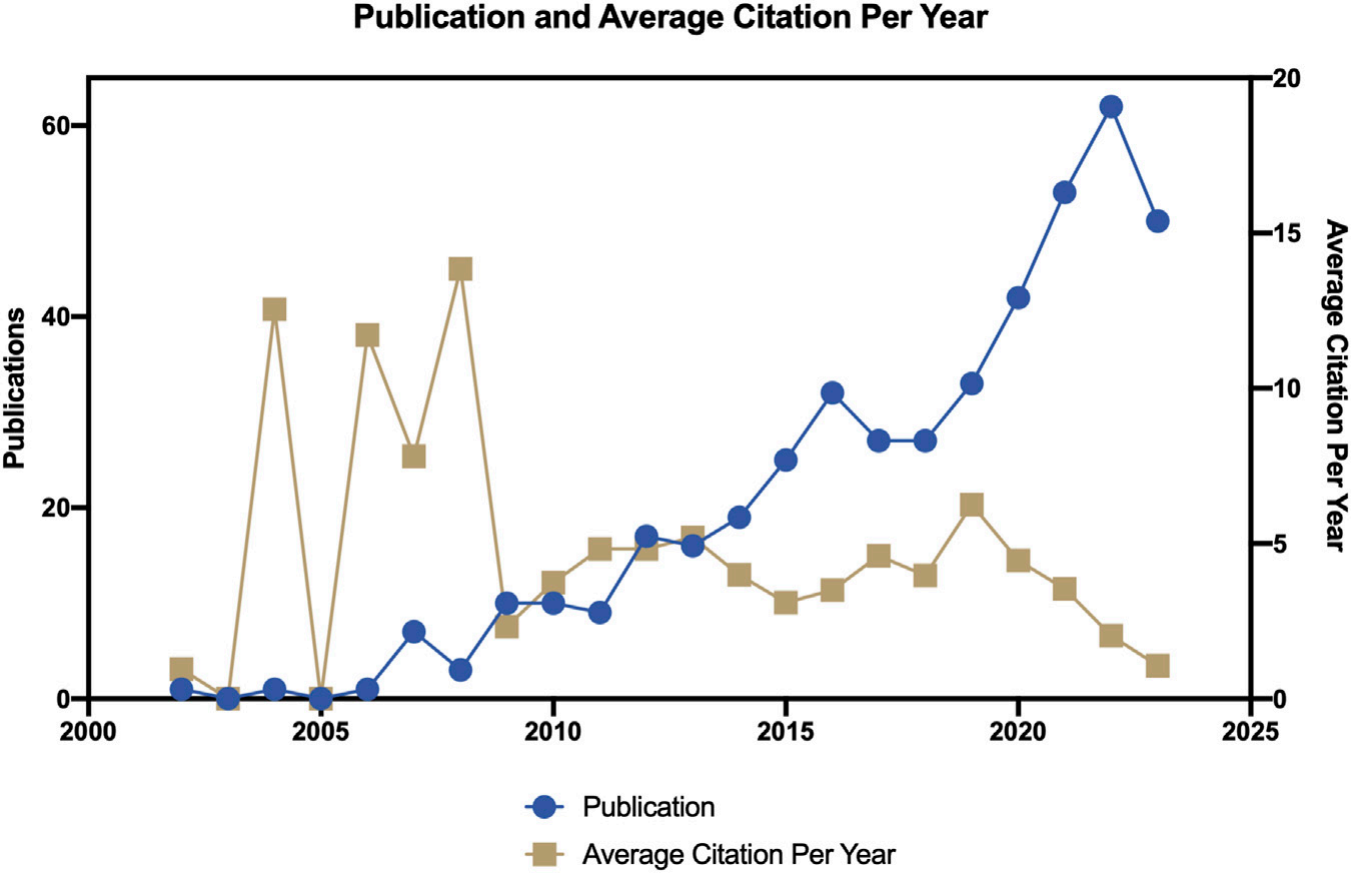
Annual output of nanotechnology in SCI.

Based on our search and literature inclusion process (Figure 1), a total of 422 publications were included in our study, of which 381 were articles and 41 were reviews. In the timespan of this research field from 2002 to 2023, a total of 2,293 authors from 155 countries and regions belonging to 1,582 organizations participated in research in this field. Related research was published in 206 journals, and the rate of international cooperation was 28.67%, with each article being cited an average of 26.78 times. As shown in Figure 2, since 2002, this field has gradually attracted the attention of researchers. After 2018, the number of published articles has increased rapidly, with an average annual growth rate of 19.75%. 2022 will be the year with the largest number of published articles, reaching 61 articles. The 422 articles in this field received a total of 10,788 citations, and the average citations per article were 26.93. Conducting an in-depth analysis of the top 100 cited documents, we found that these documents accounted for 74.8% of the total citations.

### 3.2 Country analysis

**FIGURE 3:**
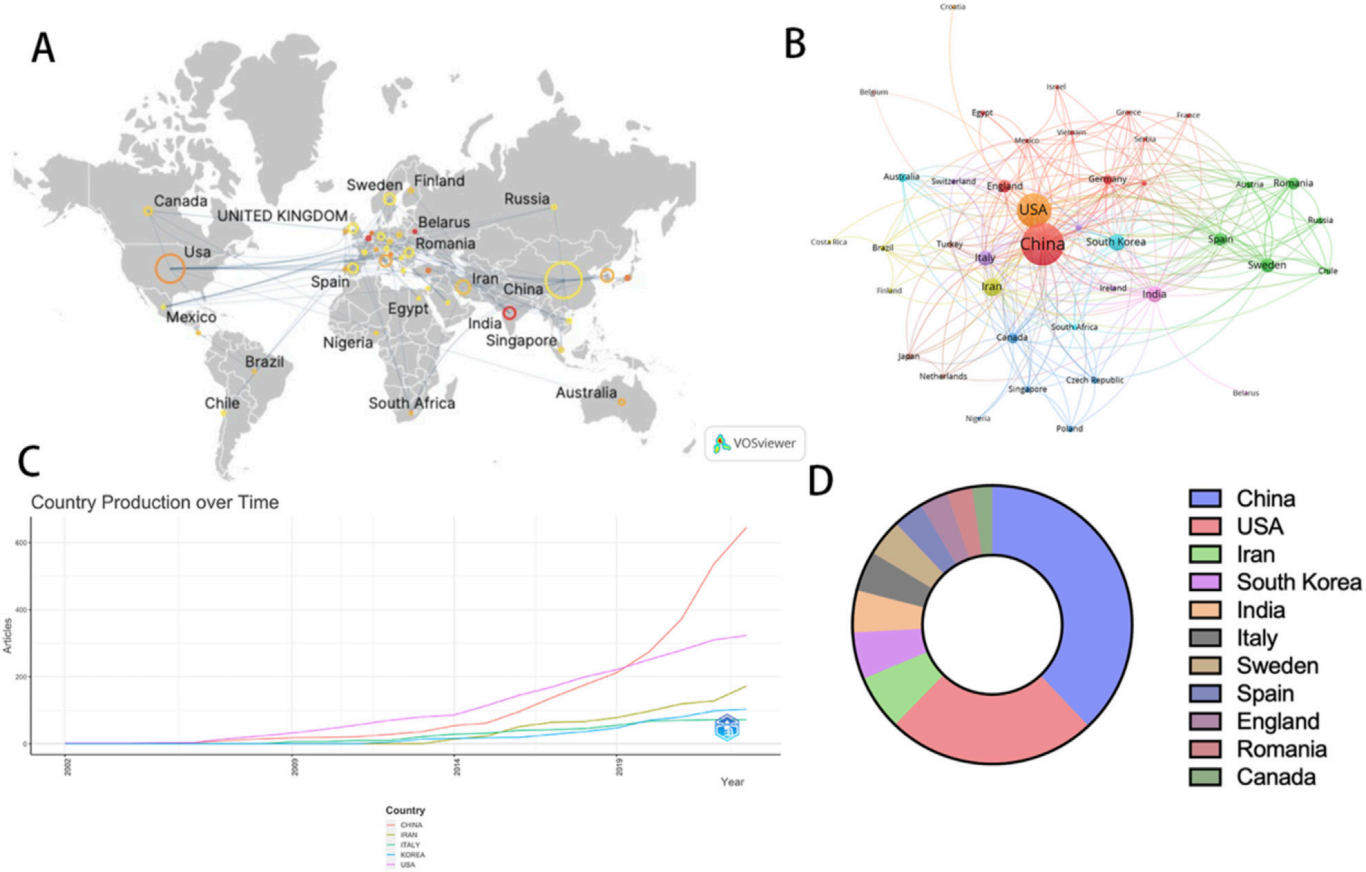
The visualization of countries on research of nanotechnology in SCI. **(A, B)** Top 5 countries by number of publications over time **(C)** Proportion of articles published by the top 10 countries **(D)**.

China and the United States are the countries with the largest number of publications in this field. Figures 3A,B illustrate the global publication situation and cooperation between countries. China published 195 articles, followed by the United States with 125 articles. In particular, in 2019, the volume of articles published by China surged rapidly, closely followed by the United States (Figure 3C). Figure 3D demonstrates the proportion of the number of articles published by the top 10 countries. It is worth noting that although the United States does not have the highest number of publications, articles from the United States receive the highest number of citations. The Average Citation Per Publication represents the level of attention a published article receives in the field. In the Average Citation Per Publication category, Israel ranks first, the United States ranks 12th, and China ranks 28th (Table 1; Supplementary Table S1). This indicates that even though China has the highest number of publications, it does not receive as much attention as Israel and the United States.

**TABLE 1:** Top 10 citations country.

Country	Citations	Publications	ACPP	Rank of ACPP
United States	5,338	125	42.704	12
China	3,280	195	17.4468	28
Italy	1,032	24	43	11
Canada	962	12	80.1667	5
Sweden	915	22	41.5909	13
India	638	25	25.52	20
South Korea	569	28	20.3214	24
Iran	472	33	14.303	32
England	468	17	31.2	15
Czech Republic	424	5	84.8	4

ACPP, represents Average Citation Per Publication.

### 3.3 Organization analysis

**FIGURE 4:**
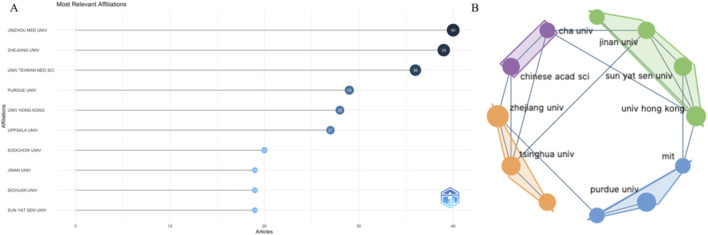
The top 10 institutions with the most publications and their cooperative relationships **(A, B)**.

A total of 1,582 organizations participated in this research area. Figure 4A shows the 10 organizations that have made the most contributions in this field, namely Jinzhou Medical University, Zhejiang University, University of Tehran Medical Science, Purdue University, University of Hong Kong, Uppsala University, Soochow University, Jinan University, Sichuan University, and Sun Yat Sen University. Among these ten organizations, seven are from China, one is from the United States, one is from Iran, and one is from Sweden. Figure 4B displays the collaboration between organizations that publish a larger number of articles. Supplementary Figure S1 illustrates the overlay situation of articles published by organizations. In the research field, early articles were primarily published by Purdue University, Northwestern University, MIT, University of Hong Kong, and other institutions, while recent articles were mainly published by the University of Chinese Academy of Science and other affiliated schools.

### 3.4 Journal analysis

A total of 206 research papers on nanotechnology in SCI were published in 18 journals that each published more than 5 papers. Figure 2 in the supplementary material shows the density visualization of journal in this field. Table 2 presents the top 10 journals with the highest number of articles. It is evident that among these top 10 journals, biomaterials have the highest number of articles, reaching up to 12. The quality of journals in this field was relatively high, with 6 journals belonging to Journal Citation Reports (JCR) Q1 and 4 journals having an impact factor of over 10.

**TABLE 2:** Top most productive journals.

Journal	Rank	Publication	JCR	Impact factor
Biomaterials	1	12	Q1	14.0
International Journal of Nanomedicine	2	12	Q1	8.0
Journal of Controlled Release	3	10	Q1	10.8
Acs Nano	4	8	Q1	17.1
Journal of Biomedical Nanotechnology	5	8	Q3	2.9
Nanomedicine-Nanotechnology Biology and Medicine	6	8	Q2	5.4
Acs Applied Materials and Interfaces	7	7	Q1	9.5
Acs Biomaterials Science and Engineering	8	7	Q2	5.8
Chemical Engineering Journal	9	7	Q1	15.1
Journal of Biomedical Materials Research Part A	10	7	Q2	4.9

### 3.5 Author analysis

**FIGURE 5:**
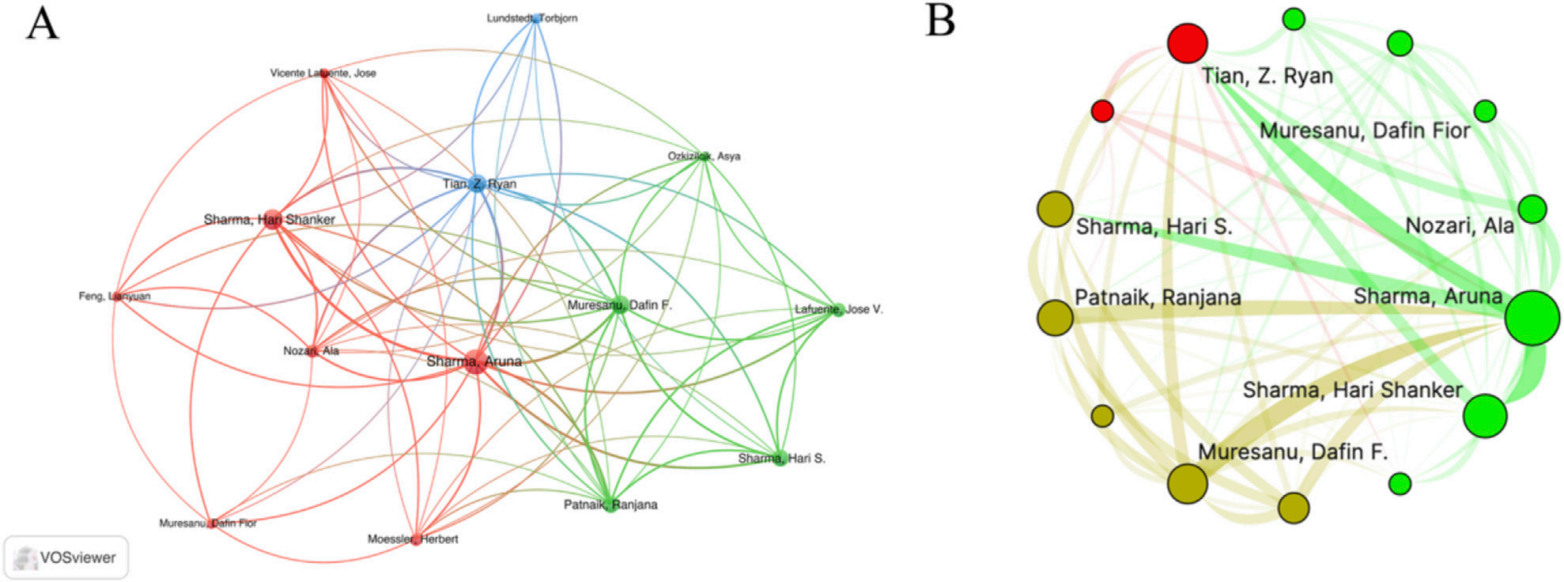
The visualization of author on research of nanotechnology in SCI **(A, B)**.

A total of 2,293 authors participated in the publication of papers in this field. Table 3 presents the top 10 authors with the highest number of published papers, namely Sharma HS, Sharma A, Muresanu DF, Lafuente JV, Mei XF, Tian ZR, Patnaik R, Forloni G, Papa S, and Rossi F. Supplementary Figure S3 shows the annual publication status of these top ten authors. It was noteworthy that the top four authors with the most published articles were all affiliated with Uppsala University in Switzerland, followed by Italy. There were two authors from the United States and China. Figure 5A and B shows the cooperation among the authors, revealing close cooperative relationships between teams from Italy, Sweden, and the United States.

**TABLE 3:** Top 10 most productive authors.

Author	Publication	H-index	Country	Affiliation
Sharma Hs	20	15	Sweden	Uppsala University
Sharma A	19	15	Sweden	Uppsala University
Muresanu Df	13	10	Sweden	Uppsala University
Lafuente Jv	11	9	Sweden	Uppsala University
Mei Xf	11	6	China	Liaoning Medical University
Tian Zr	10	8	United States	University of Arkansas
Patnaik R	9	9	Sweden	Uppsala University
Forloni G	8	8	Italy	IRCCS Istituto di Ricerche Farmacologiche “Mario Negri”
Papa S	8	8	Italy	IRCCS Istituto di Ricerche Farmacologiche “Mario Negri”
Rossi F	8	8	Italy	Materials and Chemical Engineering “Giulio Natta”

### 3.6 Analysis of the most active topics

#### 3.6.1 Subject category burst

**FIGURE 6:**
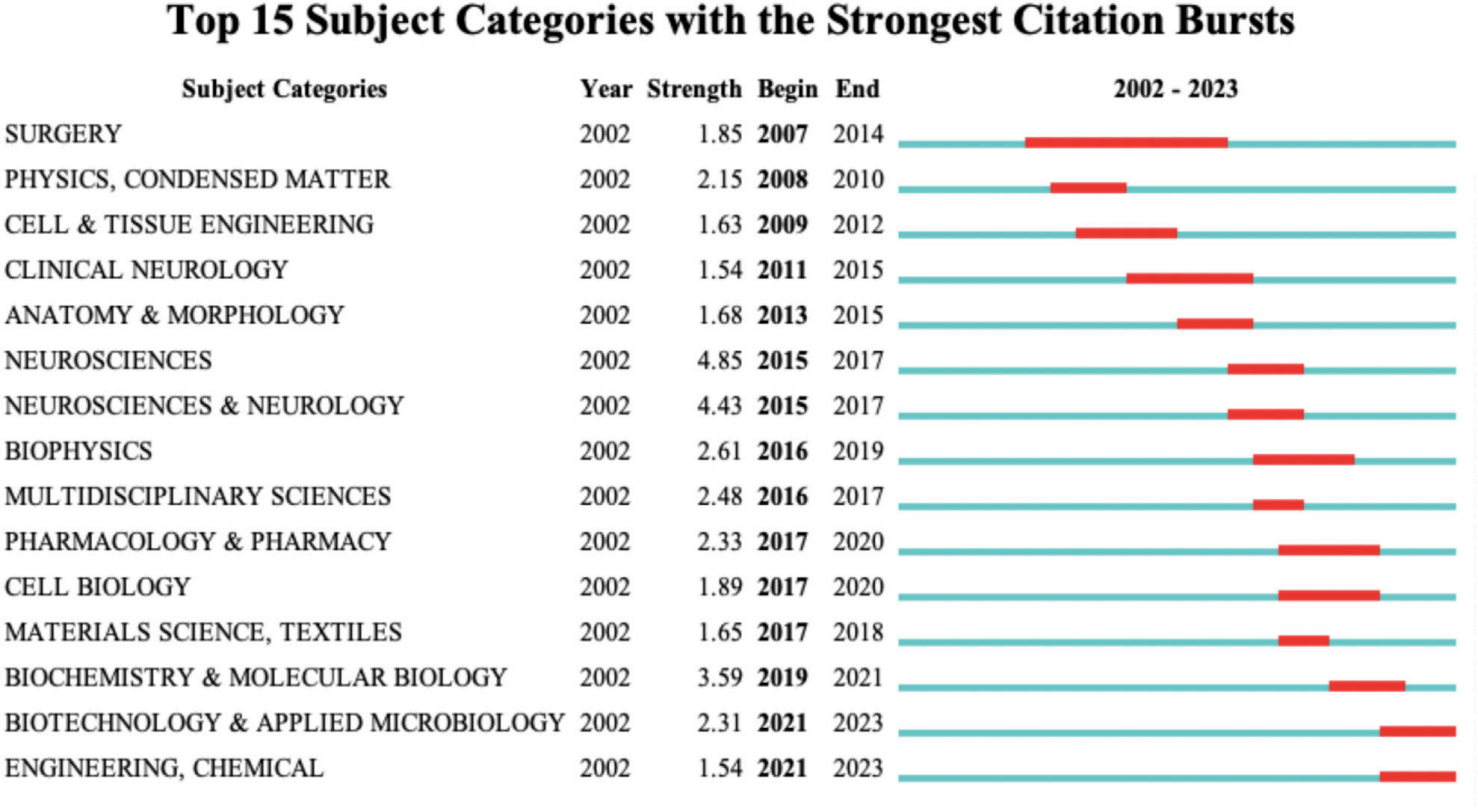
Top 15 subject categories with the strongest citation burst.

The Figure 6 displays the subject category bursts. The blue line represents the time interval, while the red line segment indicates the span of citation bursts, including the start and end years. The Figure 6 showcases the top 15 terms with the highest burst intensity during different periods. The subject category bursts evolved from “Surgery” (2007) to “Neurosciences” (2015), “Multidisciplinary Sciences” (2016), “Pharmacology and Pharmacy” (2017), “Cell Biology” (2017); and from “Physics. Condensed Matter” (2008), further developed into “Biochemistry and Molecular Biology” (2019). This progression indicates that research on nanotechnology in spinal cord injury has shifted from fields like neurosurgery and fundamental physics to disciplines such as pharmacology, cell biology, or biochemistry. For instance, the use of nanomedicine as a therapeutic approach for spinal cord injury, where Prussian blue nanocatalysts combined with zinc ions can improve the microenvironment of spinal cord injury (Gao et al., 2024); or the loading of traditional drugs (Paclitaxel, Cerebrolysin) onto nanoparticles to enhance delivery methods (Zhang et al., 2022; Sahib et al., 2020). Additionally, research on nanomaterials as carriers for cell therapy, such as adipose-derived stem cells loaded in nanogels to improve motor function after spinal cord injury (Li J. et al., 2022).

#### 3.6.2 Keyword burst

**FIGURE 7:**
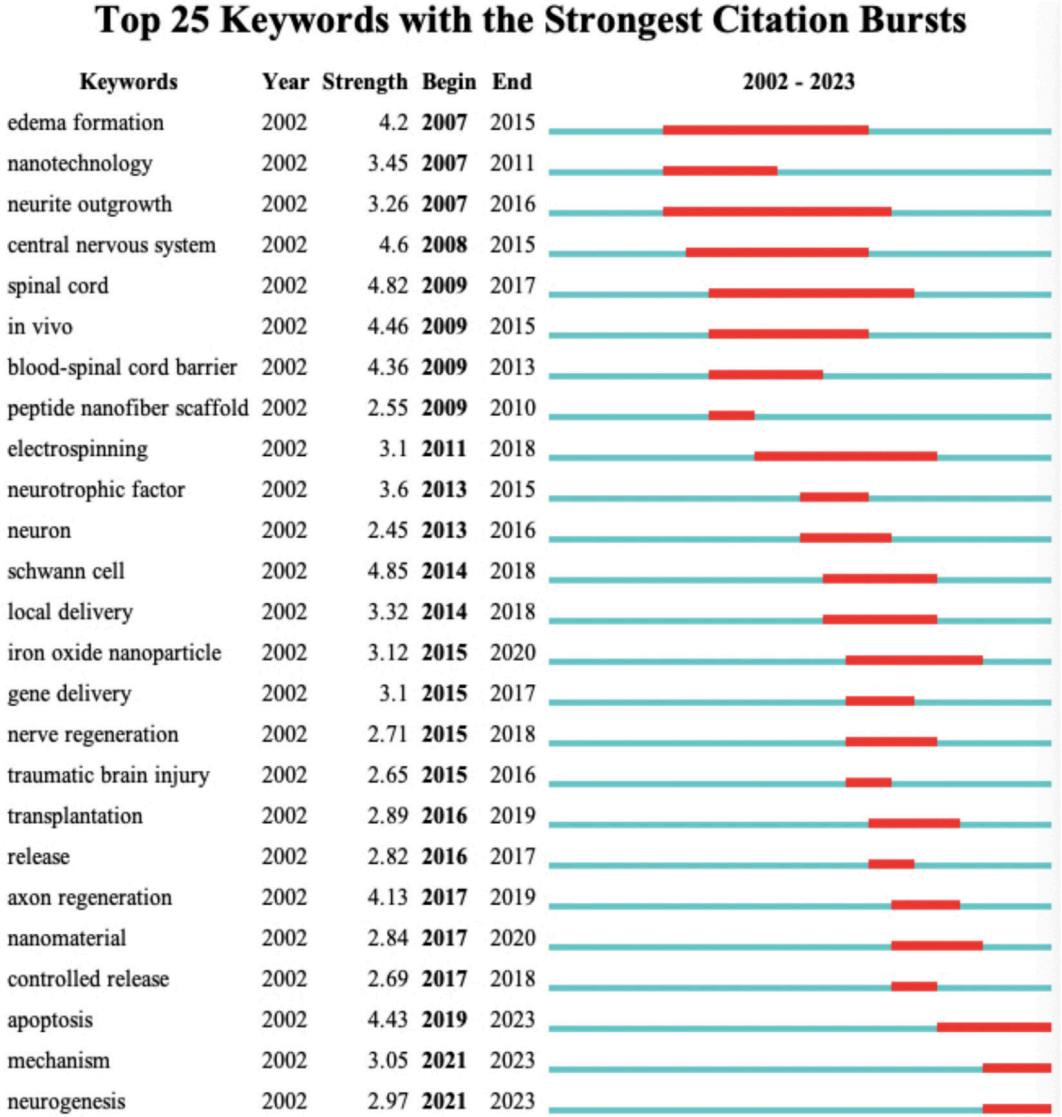
Top 25 keywords with the strongest citation bursts.

At a more refined level, we have revealed the research trends in the field of nanotechnology for spinal cord injury throughout the entire timespan (2002–2023) by examining keyword bursts. As shown in the Figure 7, the top 25 keywords with the highest degree of burst are displayed. “Edema Formation” is the earliest keyword burst, persisting from 2007 to 2015, reaching a burst value of 4.2. This indicates that during this phase, researchers primarily focused on the role of nanotechnology in eliminating spinal tissue edema following spinal cord injury. “Blood-Spinal Cord Barrier” reached a burst value of 4.37 from 2009 to 2013, signifying that at this stage, using nanotechnology to breach the blood-spinal cord barrier and enable therapeutic agents to reach the injury site was a hot research topic. In addition to the blood-spinal cord barrier, several other hotspots were observed in the keyword bursts, which I categorize into: tissue engineering material types, target cell types, tissue engineering material properties, and biological behaviors. “Peptide Nanofiber Scaffold,” “Electrospinning,” and “Iron Oxide Nanoparticle” reached bursts in 2009–2010, 2011–2018, and 2015–2018, respectively. These three types of nanomaterials for tissue engineering were among the more extensively studied material categories at the time. “Neuron” and “Schwann Cell” were keywords that burst in 2013 and 2014, respectively, indicating that these two types of cells received significant attention from researchers around 2013–2018. Keywords related to the properties of tissue engineering materials, such as “Local Delivery,” “Gene Delivery,” “Transplantation,” and “Controlled Release,” experienced bursts during the 2015–2018 period. Additionally, cellular biological behaviors like “Axon Regeneration,” “Apoptosis,” and “Neurogenesis” have burst in recent years. This perspective suggests that in the initial stages, researchers focused on the application of nanotechnology in spinal cord injury treatment as a means to eliminate spinal edema and to penetrate the blood-spinal cord barrier. With the advancement of research techniques and shifts in thinking, the focus of researchers has gradually transitioned to studying different types of nanomaterials, targeting various cells, and utilizing the advantageous properties of multiple materials to achieve the goal of improving cellular biological behaviors.

#### 3.6.3 Keyword clustering analysis

**FIGURE 8:**
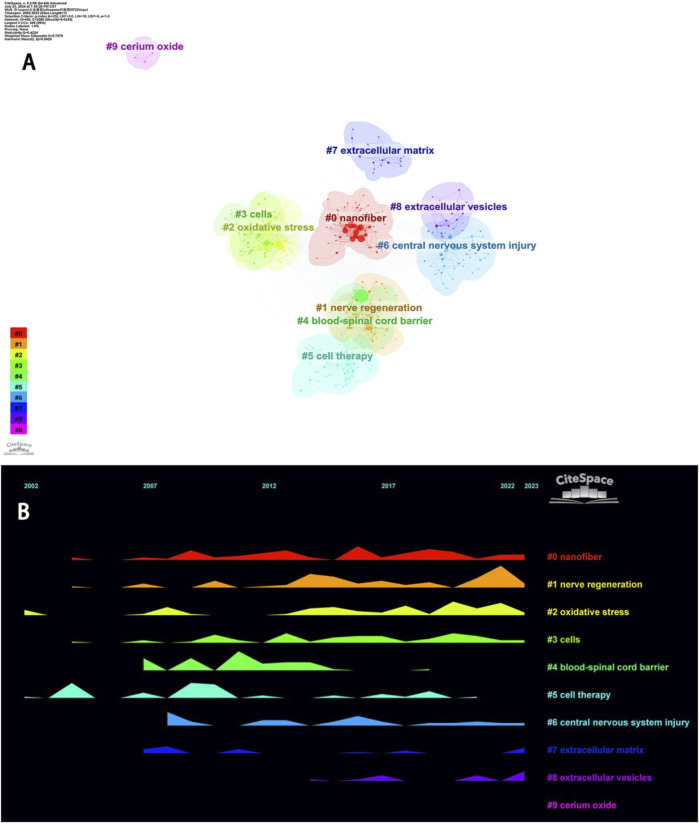
Keyword cluster snapshots. **(A)** Clustering form. **(B)** Timeline form.

Keywords exhibit strong intrinsic correlations, and certain keywords can form different clusters based on their affinity. Identifying these clusters can more intuitively delineate the various subfields of nanotechnology research in spinal cord injury. As shown in Figure 8A, keyword clustering can be categorized into four groups: different nanomaterials (#0 Nanofiber, #9 Cerium Oxide), cell-related therapies (#3 Cells, #5 Cell Therapy, #7 Extracellular Matrix, #8 Extracellular Vesicles), therapeutic targets (#2 Oxidative Stress, #4 Blood-Spinal Cord Barrier), and therapeutic goals (#1 Nerve Regeneration) as well as #6 Central Nervous System Injury. #0 Nanofiber and #6 Central Nervous System Injury are the main themes in this field, while #1 Nerve Regeneration is the goal of all research, and thus these three keyword clusters are present throughout almost the entire research process. #2 Oxidative Stress is a key pathophysiological process in secondary injury following spinal cord injury. Treatments targeting oxidative stress have been a hot research topic in recent years. #4 Blood-Spinal Cord Barrier is a major limitation for traditional pharmacological treatments of spinal cord injury. Large molecular drugs are unable to penetrate the blood-spinal cord barrier, but nanomedicines, after years of development, have demonstrated improved capabilities to cross this barrier (Khan et al., 2020; Xie et al., 2023). In recent years, traditional nanofibers for spinal cord injury treatment have shown certain limitations. However, #7 Extracellular Matrix, #8 Extracellular Vesicles, and the novel #9 Cerium Oxide nanoparticles have shown greater therapeutic significance for spinal cord injury (Figure 8B).

The *Y*-axis represents the frequency of occurrence.

### 3.7 Analysis of most cited and co-cite articles

Citation analysis is a method utilized to analyze the influence of a paper. The greater the number of citations a paper receives, the higher its influence within the academic world. Supplementary Table S2 presents the top 10 most cited articles. All ten articles have garnered over 100 citations, with the first and second-ranked articles amassing more than 500 citations. In this field, there were a total of 18,357 documents, out of which 17 have been cited more than 20 times (Supplementary Table S3).

## 4 Discussion

### 4.1 General information and knowledge base

Research on nanotechnology in SCI has primarily progressed through three stages. The first stage spanned from 2002 to 2009, marking the nascent phase of the field, characterized by a scattering of published articles. The second stage was from 2010 to 2018. The number of papers published in this stage increased slowly. The third stage, from 2019 to the present, has witnessed a rapid surge in publications (Figure 2). In this domain, China and the United States, two of the most technologically advanced countries in the world, have the highest number of publications globally. Notably, the volume of papers from China has grown swiftly after 2019, surpassing that of the United States. However, the most prolific and influential team in this field was from another country Sweden and affiliated with Uppsala University, including prominent members such as Sharma Hs, Sharma A, Muresanu Df, Lafuente Jv, and Patnaik R, et al. In this field, the most cited paper was “Auto-catalytic ceria nanoparticles offer neuroprotection to adult rat spinal cord neurons,” published by Das et al. (2007). This paper discussed the potent antioxidant and neuroprotective properties of auto-catalytic ceria nanoparticles, which could prevent ischemic SCI and offer neuroprotective effects, showing promising application potential in SCI treatment. The second most cited paper was “Self-assembling nanofibers inhibit glial scar formation and promote axon elongation after spinal cord injury “ by Tysseling-Mattiace et al. (2008). This research focused on self-assembling peptide amphiphile nanofibers, which could inhibit the differentiation of neural stem cells into glial cells and promote the regeneration of neural axons. *In vivo*, these nanofibers could reduce astrocytes and increase the proportion of oligodendrocytes. The third most cited article, “Magnetic resonance tracking of transplanted stem cells in rat brain and spinal cord (Syková and Jendelová, 2006),” described the use of iron oxide nanoparticles as markers for stem cell therapy in SCI and brain injury. The fourth-ranked paper “Nano hemostat solution: immediate hemostasis at the nanoscale (Ellis-Behnke et al., 2006),” which introduced a novel nanopeptide capable of stopping bleeding in internal organs, including the spinal cord. The fifth-ranked article, “Reknitting the injured spinal cord by self-assembling peptide nanofiber scaffold (Guo et al., 2007),” presented a self-assembled nanofiber scaffold that served as a growth platform for neural progenitor cells and Schwann cells, providing a three-dimensional environment for axon growth, with transplantation into SCI sites promoting functional recovery. The article ranked 6th was “Squalenoyl adenosine nanoparticles provide neuroprotection after stroke and spinal cord injury (Gaudin et al., 2014),” which showed that squalenoyl adenosine nanoparticles could have neuroprotective effects on rats with SCI and stroke, indicating promising application prospects. The seventh-ranked article, “Axonal alignment and enhanced neuronal differentiation of neural stem cells on graphene-nanoparticle hybrid structures (Solanki et al., 2013),” discussed the combined effect of graphene nanoparticles on the differentiation of neural stem cells. The structural characteristics of graphene can affect axonal arrangement, while nanoparticles can promote the differentiation of neural stem cells. The article ranked 8th was “Nanoparticle-mediated local delivery of Methylprednisolone after spinal cord injury (Kim et al., 2009).” Methylprednisolone pulse therapy has been the main method for the treatment of SCI, but high-dose injections may cause side effects in other organs. This paper introduced a new type of methylprednisolone nanoparticle that could be locally injected at SCI site, slowly releasing the drug for improved efficacy over systemic high-dose shock therapy and minimizing systemic side effects. The article ranked 9th was “Flexible and stretchable nanowire-coated fibers for optoelectronic probing of spinal cord circuits (Lu et al., 2017),” which covered a nanofiber capable of detecting spinal cord electrical signals during rehabilitation exercises for SCI. The article ranked 10th was Transplantation of Nanostructured Composite Scaffolds Results in the Regeneration of Chronically Injured Spinal Cords (Gelain et al., 2011). After SCI, large cavities and cysts might appear in the injured area, and nerve axons could not pass through the large cystic area. This article described a system comprising electrospun fibers and peptides. Transplanting this nano-drug-delivery system into the SCI area could result in re-covering of the spinal cord cyst area with a nerve cell nuclear matrix, significantly enhancing overall motor function scores.

Co-cited articles indicated that two articles were cited together by other articles. Commonly cited articles represented the foundational articles and knowledge background in this field. Supplementary Table S3 shows the top ten papers with the highest number of common citations. Articles ranked 2 (Kim et al., 2009) and 4 (Tysseling-Mattiace et al., 2008) functioned as highly cited articles, which were introduced in the previous section. Among these ten articles, four articles simply introduced information related to SCI. One article introduced the Basso-Beattie-Bresnahan (BBB) score, which is the most commonly used scoring method for rat SCI models. This article also has the highest number of citations (Basso et al., 1995). Three articles respectively discussed the epidemiology, pathological changes, prognosis (Ahuja et al., 2017), treatment methods (Thuret et al., 2006), and targets (Silver and Miller, 2004) of SCI. Additionally, there were two articles that introduce the application and therapeutic effect of combining methylprednisolone, a commonly used drug for SCI, with nanoparticles in SCI. These studies found that sustained release of methylprednisolone could lead to better therapeutic effects (Chvatal et al., 2008; Cerqueira et al., 2013). The remaining two articles describe the potential application of two different nanomaterials in SCI (Silva et al., 2004; Shi et al., 2010).

### 4.2 Frontiers and hotspot

The inflammatory response following SCI was characterized by several key events.

Recruitment of immune cells: Following an injury, immune cells such as macrophages and neutrophils are promptly attracted to the site of damage. These immune cells play a crucial role in clearing debris and pathogens, but their activation can also contribute to inflammation (Brockie et al., 2024; Lv et al., 2024; Li C. et al., 2022). Release of pro-inflammatory cytokines tumor necrosis factor (TNF)-α (Cao et al., 2022), interleukin (IL)-1β (Müller et al., 2023), and IL-6 (Shipman et al., 2023), were released as part of the immune response. These cytokines can activate intracellular signaling pathways, including those involved in apoptosis (Ding et al., 2023). Activation of signaling pathways: The inflammatory environment can activate specific signaling pathways within cells. For example, TNF-α has been known to engage the extrinsic apoptotic pathway, leading to cell death (Zhou et al., 2021). This activation of apoptotic pathways can result in the loss of neurons and other essential cells within the spinal cord, thereby exacerbating the severity of the injury (Weng et al., 2023).

Conversely, apoptosis itself could trigger or intensify the inflammatory response through various mechanisms. Release of damage-associated molecular patterns (DAMPs): When cells undergo apoptosis, they release intracellular components and molecules known as DAMPs. DAMPs act as danger signals, alerting the immune system to the presence of cell damage or death (Lyu et al., 2023). Immune cell activation: DAMPs released during apoptosis can activate nearby immune cells, such as dendritic cells and macrophages. This activation stimulates the immune cells to release pro-inflammatory cytokines and chemokines, further promoting an inflammatory environment (Li and Lan, 2023). Amplification of inflammation: The continued presence of DAMPs and the release of pro-inflammatory factors can exacerbate the inflammatory milieu at the injury site (Das et al., 2024). This leads to the sustained recruitment of more immune cells to the site and the perpetuation of the inflammatory response, potentially causing further tissue damage.

The interplay between apoptosis and inflammation in SCI was a complex and dynamic process. Inflammatory triggers can induce apoptosis in damaged cells, contributing to tissue loss. Conversely, apoptosis can stimulate an inflammatory response by releasing danger signals, leading to the recruitment of more immune cells and the persistence of inflammation. Understanding these mechanisms is crucial for developing targeted therapeutic interventions, including nanotechnology approaches, to modulate this crosstalk and promote improved outcomes in patients with SCI.

Nanotechnology has shown promise in modulating apoptosis and inflammation in the SCI process (Luo W. et al., 2023). Nanotechnology, which harnesses the power of nanoparticles for therapeutic purposes, holds great potential in modulating the intricate interplay between apoptosis and inflammation following SCI. Jaffer et al. (2023) found that injecting antioxidant nanoparticles into the spinal cord injury site activated microglia to differentiate in an anti-inflammatory direction. This process inhibited nerve cell apoptosis in the injured area.

Nanoparticles can be meticulously designed and customized to transport a wide array of therapeutic agents, ranging from drugs (Yin et al., 2023) and growth factors (Xia et al., 2021; Zhu et al., 2016) to anti-inflammatory compounds. The CC-chemokine ligand 2 (CCL2) - CC-chemokine receptor 2 (CCR2) axis is an inflammatory therapeutic target in SCI (Geng et al., 2022). Encapsulating drugs in the cell membrane of macrophages overexpressing CCR2 can promote targeted delivery of drugs to the injured area and reduce the infiltration of inflammatory cells and neuronal apoptosis (Gu et al., 2023). One of the difficulties in treating SCI is the efficiency with which drugs cross the blood-spinal cord barrier. Improving drug penetration into the blood-spinal cord barrier is one of the current design concepts of nanotechnology. R2KC peptide (Wu et al., 2023), and choline (Bao et al., 2023) are applied to the surface of nanotechnology to enhance their ability to penetrate the blood-spinal cord barrier. Click chemistry is applied in the preparation of nanotechnology. By incorporating peptides such as isoleucyl-lysyl-valyl-alanyl-valine (IKVAV) (Zeng et al., 2023) and DA7R (Ruan et al., 2023) onto the surface of exosomes derived from M2 macrophages using click chemistry methods, this novel nano-preparation can be targeted and transported to the damaged area to reduce the inflammatory response in the affected region. This tailored approach enables the precise delivery of these agents to the injured site within the spinal cord, thereby minimizing off-target effects and significantly enhancing therapeutic efficacy.

In the context of SCI, specific nanoparticles have been engineered to serve as powerful allies. They can act as scavengers of inflammation, effectively neutralizing pro-inflammatory molecules and modulating the behavior of immune cells. In the current study, drugs such as curcumin (Xiong W. et al., 2023), metformin (Su et al., 2023; Liu X. et al., 2023), and rapamycin (Shen et al., 2023) were encapsulated in nanomaterials. This drug delivery system further promotes the polarization of macrophages towards the M2 phenotype and reduces the infiltration of inflammatory factors related to the injured area. In addition to metallic nanomaterials, exosomes have also been used to deliver anti-inflammatory drugs. Incorporating non-coding RNAs such as miR-672-5p (Zhou et al., 2022) or specific proteins (Li et al., 2020) into exosomes, or overexpressing certain proteins like LRRC75A-AS1 (Xing et al., 2024), miR-216a-5p (Xue et al., 2023) miR-9-5p (He et al., 2022), miR-137 (Shao et al., 2023), and other methods, can improve inflammatory symptoms in SCI areas. Meanwhile, Zinc Oxide and tubastatin are dedicated to promoting cell survival and stimulating tissue regeneration (Lin et al., 2021; Liao et al., 2022). These nanoparticles deliver neuroprotective factors and facilitate the repair of damaged tissues, helping to counteract both apoptosis and inflammation.

**FIGURE 9:**
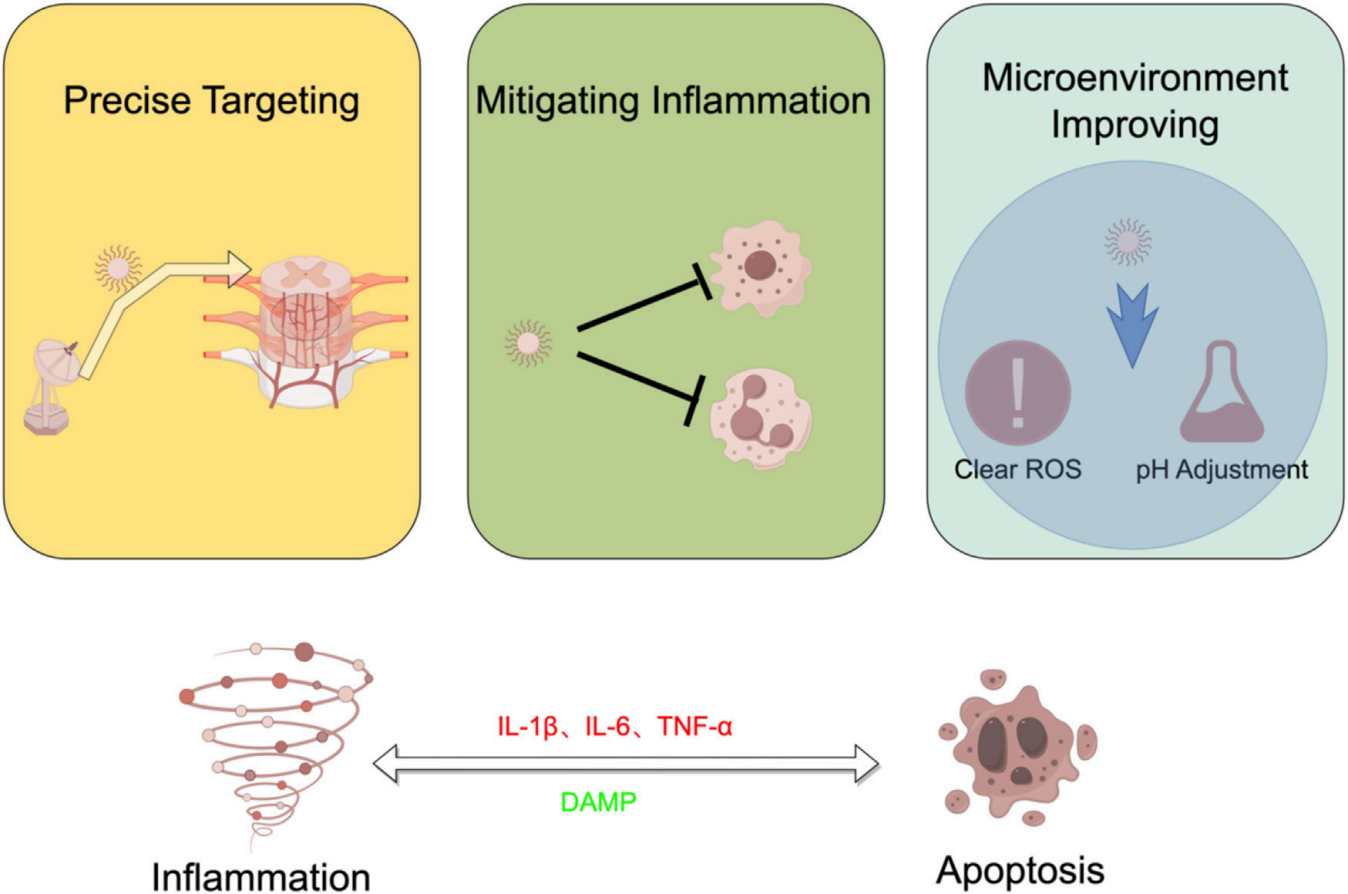
Hot spots and frontiers in the field of nanotechnology in SCI research in recent years.

One of the remarkable attributes of nanotechnology is its adaptability. Nanoparticles can be finely tuned to respond to specific cues within the injury microenvironment. For instance, they can be designed to release therapeutic agents in response to indicators of inflammation or changes in pH levels. The nano-drug delivery system of chitosan and poly acrylic acid designed by Sabourian et al. (2023) can decompose in the acidic environment of the injured area, allowing the drug to enter the cell nucleus and exert a regulatory effect. Qian et al. (2023) synthesized curcumin on pH-responsive nanomicelles, which can be released in the damaged area to exert anti-inflammatory effects. This dynamic responsiveness helps maintain a more conducive and supportive environment for the healing process, addressing the challenges posed by both apoptosis and inflammation. In the microenvironment of SCI, high concentrations of reactive oxygen species (ROS) severely limit the therapeutic effect of drugs. Removing ROS from the microenvironment is an important therapeutic approach. Liu et al. (Liu D. et al., 2023) applied cerium oxide nanoparticles to the treatment of SCI and found that this treatment can reduce ROS in the environment and improve the survival and differentiation of transplanted neural stem cells. Ma et al. (2023) used the ROS scavenging effect of Polydopamine to improve the efficacy of neurotrophic factors in the injured area. Nanozymes have attracted significant attention due to their unique structure and catalytic effect in improving the microenvironment of SCI areas. Shen et al. (2023) used Prussian blue nanozyme to target the SCI area, effectively clearing ROS while improving inflammation and apoptosis-induced neural damage. Xiong T. et al. (2023) discovered that the nanozyme Mn3O4 has a higher substrate affinity than ordinary antioxidant enzymes and shows superior ROS scavenging effects in SCI Figure 9.

Nanotechnology emerges as a promising avenue for tackling the complex interplay between apoptosis and inflammation in SCI. By offering precise targeting, inflammation mitigation, and promotion of the microenvironment, nanotechnology stands at the forefront of innovative approaches to enhance recovery and reduce the impact of these interconnected processes.

In addition to studying the effects of different nanotechnology on SCI, the mechanism of drug action is also a research focus in this field. Inflammatory signaling pathways are crucial targets for the treatment of SCI. Macrophage polarization can be regulated by inhibiting these pathways. Sesamol-loaded nanomicelles have been shown to inhibit apoptosis and pro-inflammatory factors through the NF-κB signaling pathway (Wang N. et al., 2021). Human umbilical cord mesenchymal stem cells target TLR4/NF-κB p65 to inhibit IL-1β, IL-6, TNFα and other cytokines (Wang et al., 2023). Stem cell exosomes can promote microglial M2 polarization by activating the nuclear factor erythroid 2-related factor 2 (Nrf2)/heme oxygenase-1 (HO-1) (Luo Y. et al., 2023) and suppressor of cytokine signaling 3 (SOCS3)/Janus Kinase 2 (JAK2)/signal transducer and activator of transcription 3 (STAT3) (Yuan et al., 2023) inflammatory signaling pathways, thereby promoting neurological recovery. Angiogenesis is another important target for treating SCI. New blood vessels can provide necessary nutrients for repairing the injured area. Exosomes in cerebrospinal fluid can promote angiogenesis in the injured area through activation of the phosphoinositide 3-kinase (PI3K)/protein kinase B (AKT) signaling pathway (Li et al., 2023),while exosomes derived from M2 macrophages can promote vascular regeneration through the hypoxia-inducible factor (HIF)-1α/vascular endothelial-derived growth factor (VEGF) signaling pathway (Huang et al., 2022). TGF-betaR2 (Zhu et al., 2021) and nerve growth factor (NGF)/tropomyosin receptor kinase A (TrkA) (Zhao et al., 2021) are important targets for nanotechnology in promoting nerve regeneration.

## 5 Strength and limitation

This study had the following advantages: (1) It was the first bibliometric study on nanotechnology in SCI research, providing a systematic and comprehensive overview of the current research status in this field. It highlighted the most important articles, the most active research teams, and current research hotspots.; (2) It provided a visually appealing and easily understandable summary in the form of a picture. However, this study also had certain limitations: (1) Only English-language documents were included, potentially excluding relevant studies published in other languages. Some documents may have been unintentionally missed during the study selection process. (2) Although there is a lot of basic research in this field, clinical trials are still rare. Some clinical trials have found that the combination of stem cells and scaffolds has good effects in patients with spinal cord injuries (Xiao et al., 2018). More clinical research is needed in the future to enrich this field.

## 6 Conclusion

This bibliometric analysis provided a comprehensive overview of the significant progress achieved in nanotechnology research, specifically within the field of SCI. The findings demonstrated a considerable increase in global research output, particularly in the past decade. Furthermore, the analysis revealed a collaborative and geographically diverse research landscape, with noteworthy contributions from institutions in China, the United States, and Europe. The presence of certain organizations and authors indicated a solid knowledge base, while the examination of citations highlighted the influential works that have shaped current trends. Within the realm of SCI treatment, the intersection with nanotechnology was characterized by a specific focus on addressing inflammation and apoptosis. Research efforts were primarily directed towards the development of nanotechnology capable of precise targeting, inflammation mitigation, and promotion of a conducive microenvironment for healing. Despite certain limitations, such as the restriction to English-language documents, the trends and insights of this study offered a valuable framework for future research directions, emphasizing the need for continued innovation and interdisciplinary collaboration to overcome the challenges presented by SCI. Future research should pay more attention to the following: How to combine nanomaterials with new treatment methods such as gene therapy and stem cell therapy, how to further improve the targeting and therapeutic efficiency of nanomedicines, the long-term biocompatibility of nanomaterials, the control mechanism of drug release, and the obstacles to large-scale clinical applications.

## Data Availability

The raw data supporting the conclusions of this article will be made available by the authors, without undue reservation.
