# Supplementary material for: Bibliometric analysis of nanotechnology in spinal cord injury: current status and emerging frontiers
Source: Front Pharmacol. 2024 Dec 11;15:1473599. doi: 10.3389/fphar.2024.1473599 (PMC11668783; doi:10.3389/fphar.2024.1473599)
Supplement: Supplementary file 1 [file Table1.docx]

Supplementary Table 1: TOP 30 ACPP Country

| Rank | Country | ACPP | Citations | Publications |
| --- | --- | --- | --- | --- |
| 1 | Israel | 146 | 146 | 1 |
| 2 | France | 103 | 206 | 2 |
| 3 | Switzerland | 101.3333 | 304 | 3 |
| 4 | Czech Republic | 84.8 | 424 | 5 |
| 5 | Canada | 80.1667 | 962 | 12 |
| 6 | Singapore | 70.4 | 352 | 5 |
| 7 | Turkey | 54.5 | 218 | 4 |
| 8 | Greece | 53 | 53 | 1 |
| 9 | Serbia | 53 | 53 | 1 |
| 10 | Japan | 48.2 | 241 | 5 |
| 11 | Italy | 43 | 1032 | 24 |
| 12 | USA | 42.704 | 5338 | 125 |
| 13 | Sweden | 41.5909 | 915 | 22 |
| 14 | Belarus | 32 | 32 | 1 |
| 15 | England | 31.2 | 468 | 17 |
| 16 | Austria | 28.4 | 142 | 5 |
| 17 | Netherlands | 27 | 81 | 3 |
| 18 | Portugal | 26.75 | 107 | 4 |
| 19 | Saudi Arabia | 26 | 78 | 3 |
| 20 | India | 25.52 | 638 | 25 |
| 21 | Belgium | 25 | 50 | 2 |
| 22 | Ireland | 25 | 75 | 3 |
| 23 | Chile | 24 | 96 | 4 |
| 24 | South Korea | 20.3214 | 569 | 28 |
| 25 | Romania | 19.7333 | 296 | 15 |
| 26 | Spain | 18.3889 | 331 | 18 |
| 27 | Poland | 18 | 90 | 5 |
| 28 | China | 17.4468 | 3280 | 195 |
| 29 | Russia | 17 | 85 | 5 |
| 30 | Germany | 16.3 | 163 | 10 |
