# Supplementary material for: Bibliometric analysis of nanotechnology in spinal cord injury: current status and emerging frontiers
Source: Front Pharmacol. 2024 Dec 11;15:1473599. doi: 10.3389/fphar.2024.1473599 (PMC11668783; doi:10.3389/fphar.2024.1473599)
Supplement: Supplementary file 2 [file Table2.docx]

Supplementary Table 2: Top 10 Most Cited Publications

| Paper | Citations | Title | Journal | PMID |
| --- | --- | --- | --- | --- |
| Das 2007 | 586 | Auto-catalytic ceria nanoparticles offer neuroprotection to adult rat spinal cord neurons | Biomaterials | 17222903 |
| Mattiace 2008 | 555 | Self-assembling nanofibers inhibit glial scar formation and promote axon elongation after spinal cord injury | J Neurosci | 18385339 |
| Syková 2006 | 251 | Magnetic resonance tracking of transplanted stem cells in rat brain and spinal cord | Neurodegener Dis | 16909039 |
| Ellis 2006 | 211 | Nano hemostat solution: immediate hemostasis at the nanoscale | Nanomedicine | 17292144 |
| Guo 2007 | 194 | Reknitting the injured spinal cord by self-assembling peptide nanofiber scaffold | Nanomedicine | 17964861 |
| Gaudin 2014 | 174 | Squalenoyl adenosine nanoparticles provide neuroprotection after stroke and spinal cord injury | Nat Nanotechnol | 25420034 |
| Solanki 2013 | 169 | Axonal alignment and enhanced neuronal differentiation of neural stem cells on graphene-nanoparticle hybrid structures | Adv Mater | 23824715 |
| Kim 2009 | 161 | Nanoparticle-mediated local delivery of Methylprednisolone after spinal cord injury | Biomaterials | 19185913 |
| Lu 2017 | 154 | Flexible and stretchable nanowire-coated fibers for optoelectronic probing of spinal cord circuits | Sci Adv | 28435858 |
| Gelain 2011 | 148 | Transplantation of Nanostructured Composite Scaffolds Results in the Regeneration of Chronically Injured Spinal Cords | ACS Nano | 21189038 |
