# Supplementary material for: Bibliometric analysis of nanotechnology in spinal cord injury: current status and emerging frontiers
Source: Front Pharmacol. 2024 Dec 11;15:1473599. doi: 10.3389/fphar.2024.1473599 (PMC11668783; doi:10.3389/fphar.2024.1473599)
Supplement: Supplementary file 3 [file Table3.docx]

Supplementary Table 3: Top 10 Most Co-Cited Publications

| Paper | Citations | Title | Journal | PMID |
| --- | --- | --- | --- | --- |
| Basso 1995 | 67 | A sensitive and reliable locomotor rating scale for open field testing in rats | J Neurotrauma | 7783230 |
| Kim 2009 | 45 | Nanoparticle-mediated local delivery of Methylprednisolone after spinal cord injury | Biomaterials | 19185913 |
| Ahuja 2017 | 43 | Traumatic spinal cord injury | Nat Rev Dis Primers | 28447605 |
| Tysseling-Mattiace 2008 | 29 | Self-assembling nanofibers inhibit glial scar formation and promote axon elongation after spinal cord injury | J Neurosci | 18385339 |
| Silva 2004 | 28 | Selective differentiation of neural progenitor cells by high-epitope density nanofibers | Science | 14739465 |
| Chvatal 2014 | 27 | Spatial distribution and acute anti-inflammatory effects of Methylprednisolone after sustained local delivery to the contused spinal cord | Biomaterials | 18255138 |
| Silver 2004 | 27 | Regeneration beyond the glial scar | Nat Rev Neurosci | 14735117 |
| Thuret 2006 | 27 | Therapeutic interventions after spinal cord injury | Nat Rev Neurosci | 16858391 |
| Cerqueira 2013 | 25 | Microglia response and in vivo therapeutic potential of methylprednisolone-loaded dendrimer nanoparticles in spinal cord injury | Small | 23161735 |
| Shi 2010 | 23 | Effective repair of traumatically injured spinal cord by nanoscale block copolymer micelles | Nat Nanotechnol | 19898498 |
