# Supplementary figures and images for: Bibliometric analysis of nanotechnology in spinal cord injury: current status and emerging frontiers
Source: Front Pharmacol. 2024 Dec 11;15:1473599. doi: 10.3389/fphar.2024.1473599 (PMC11668783; doi:10.3389/fphar.2024.1473599)

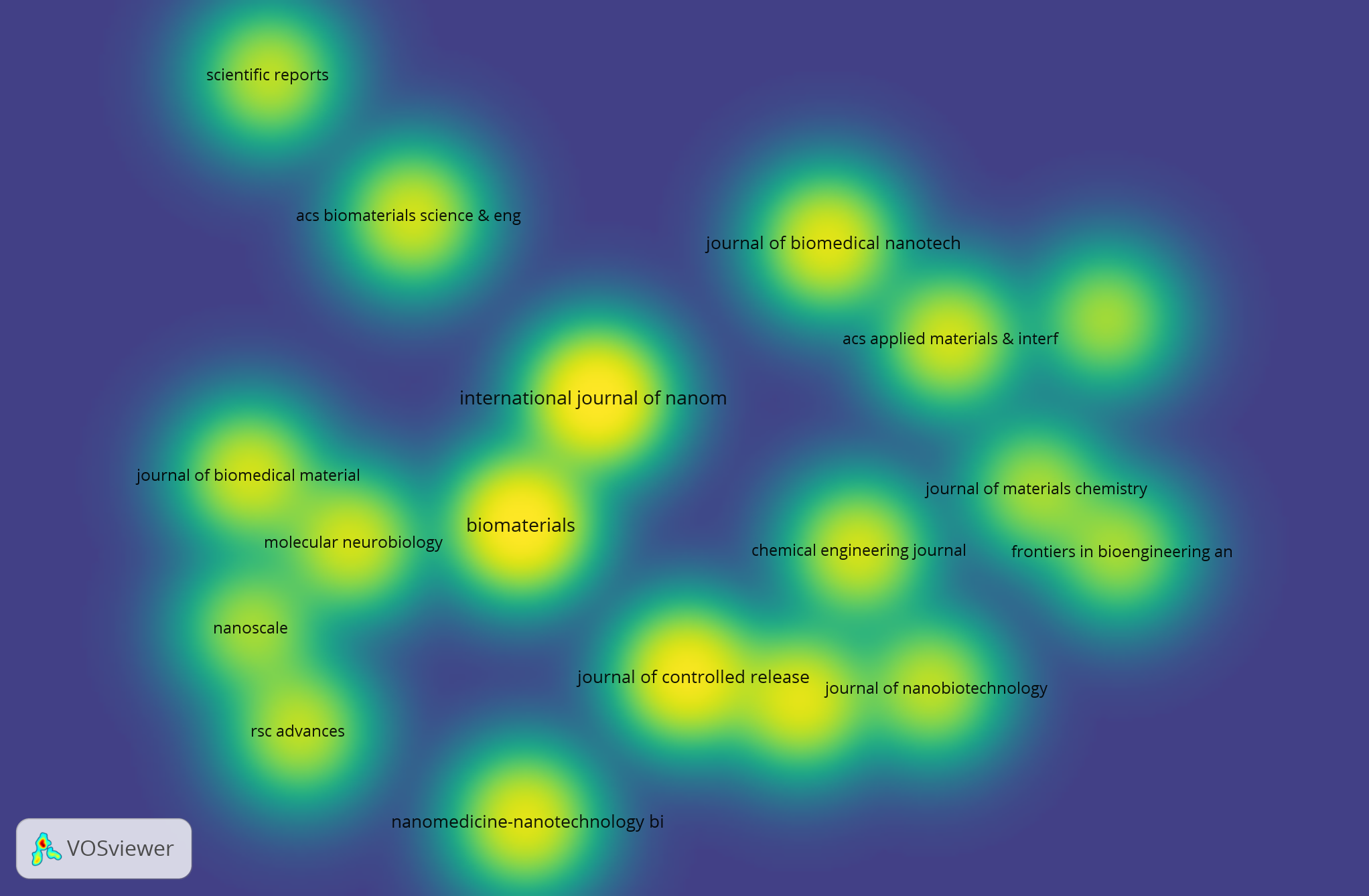

Supplement: Supplementary file 4 [file Image2.png]

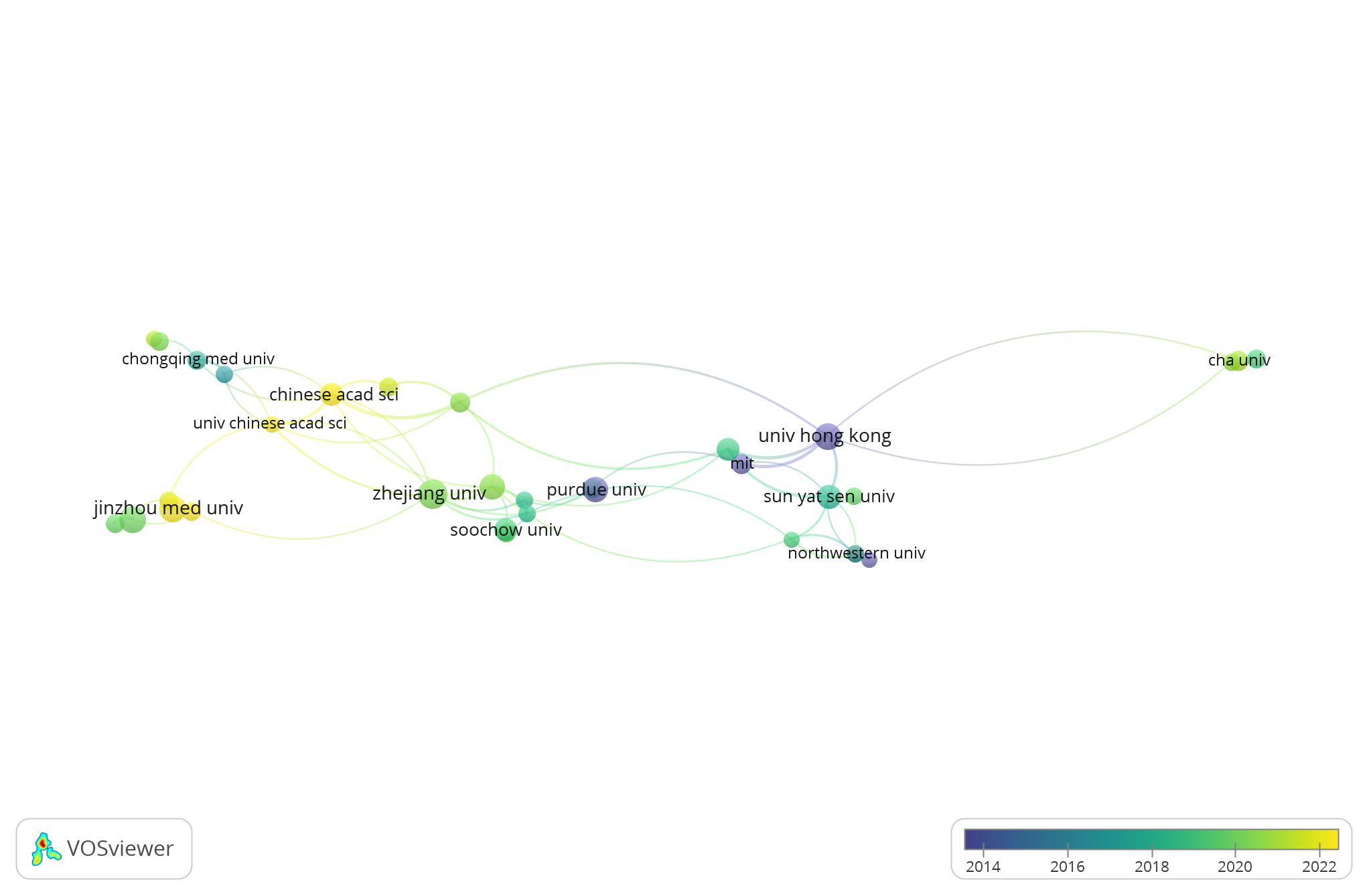

Supplement: Supplementary file 5 [file Image1.png]

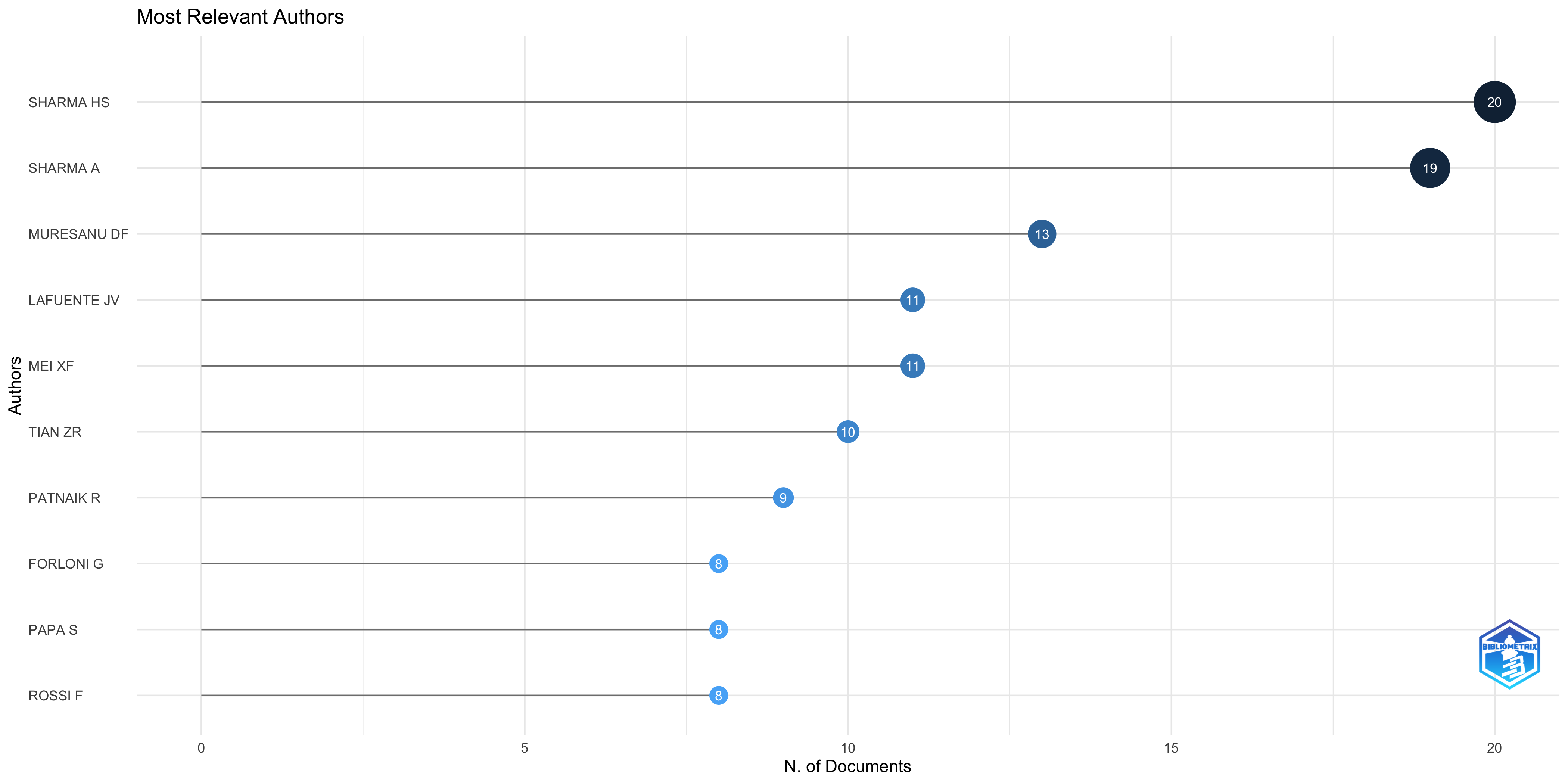

Supplement: Supplementary file 6 [file Image3.png]
